# Estimation of Human Chronological Age from Buccal Swab Samples through a DNA Methylation Analysis Approach of a Five-Locus Multiple Regression Model

**DOI:** 10.3390/ijms25020935

**Published:** 2024-01-11

**Authors:** Beatrice Marcante, Arianna Delicati, Martina Onofri, Pamela Tozzo, Luciana Caenazzo

**Affiliations:** 1 Legal Medicine Unit, Department of Cardiac, Thoracic, Vascular Sciences and Public Health, University of Padova, 35122 Padova, Italy; beatrice.marcante@phd.unipd.it (B.M.); arianna.delicati@phd.unipd.it (A.D.); pamela.tozzo@unipd.it (P.T.); 2 Section of Legal Medicine, Department of Medicine and Surgery, Santa Maria Hospital, University of Perugia, 05100 Terni, Italy; martina.onofri@live.it

**Keywords:** DNA methylation, age estimation, epigenetics, buccal swabs, forensic genetics

## Abstract

Recent advancements in forensic genetics have facilitated the extraction of additional characteristics from unidentified samples. This study delves into the predictive potential of a five-gene (*ELOVL2*, *FHL2*, *KLF14*, *C1orf132*, and *TRIM59*) methylation rate analysis for human age estimation using buccal swabs collected from 60 Italian volunteers. The methylation levels of specific CpG sites in the five genes were analyzed through bisulfite conversion, single-base extension, and capillary electrophoresis. A multivariate linear regression model was crafted on the training set, then the test set was employed to validate the predictive model. The multivariate predictive model revealed a mean absolute deviation of 3.49 years in the test set of our sample. While limitations include a modest sample size, the study provides valuable insights into the potential of buccal swab-based age prediction, aiding in criminal investigations where accurate age determination is crucial. Our results also highlight that it is necessary to investigate the effectiveness of predictive models specific to biological tissues and individual populations, since models already proven effective for other populations or different tissues did not show the same effectiveness in our study.

## 1. Introduction

Over the course of the last twenty years, advancements in forensic genetics have led to the development of techniques that empower investigators to uncover, from samples and traces of unknown origin, additional features beyond the genetic profile [1]. These innovations serve the purpose of narrowing down the number of suspects, revitalizing investigations in cold cases and aiding in the identification of unknown victims in mass casualties. Specifically, these methods focus on predicting age or particular phenotypic characteristics, such as eye, skin, and hair color, and tracing biogeographic heritage or ancestry [2].

While the analysis of particular SNP groups is predominantly employed to study phenotypic traits and biogeographical origins and has been widely studied and documented in the literature [3], human age prediction primarily relies on analyzing specific epigenetic patterns within certain genes [4,5].

**Figure 1:**
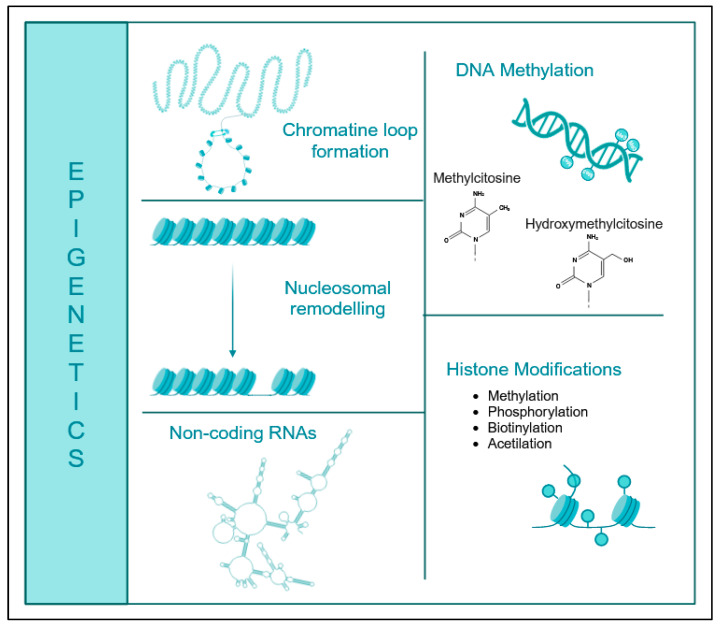
Graphic representation of the main epigenetic mechanisms. (Created with BioRender.com accessed on 20 December 2023).

Epigenetics encompass the scope of post-translational modifications occurring at the DNA or histone level and in DNA-associated chromatin proteins. These modifications can influence the accessibility of DNA and subsequently impact gene expression [6]. Epigenetics may include a range of changes, such as methylation, as detailed further, as well as acetylation, ubiquitylation, phosphorylation, sumoylation, and biotinylation [7] (Figure 1).

Alterations in the epigenetic state, particularly in the methylation patterns of specific genes, play a crucial role in regulating various phases of the cell cycle and gene expression; therefore, assessing the DNA methylation levels of diverse genes holds significant importance in numerous forensic and medical domains [8]. Considering the knowledge we have gained so far, we can state that the examination of DNA methylation patterns in forensics can unveil underlying disease conditions, shed light on factors leading to death, and aid in identifying tissue types [9,10,11]. Moreover, emerging technologies utilizing DNA methylation profiling can provide investigators with insights into the timing of and the methods involved in the deposition of traces and biological sources during illicit activities or at a crime scene [12].

**Figure 2:**
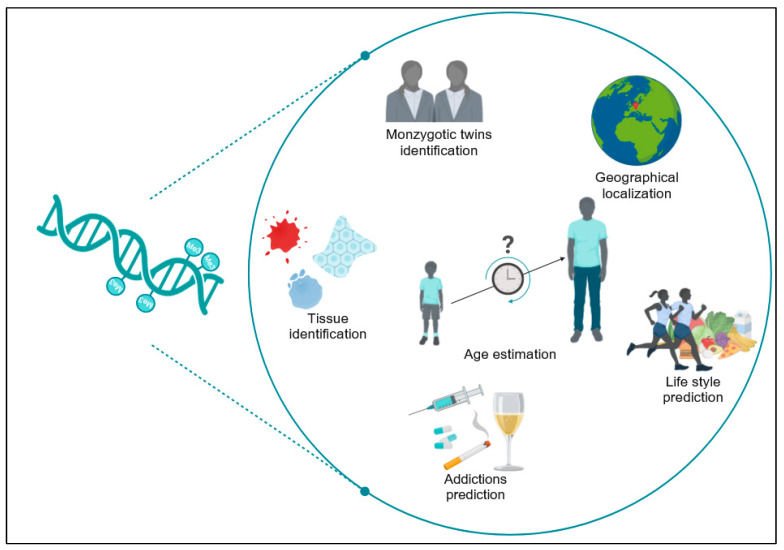
Main potential applications of epigenetics in forensics (Created with BioRender.com accessed on 20 December 2023).

As scientific advancements in human epigenomics continue to be made rapidly, the creation of an “epigenomic fingerprint” from traces at crime scenes is becoming plausible, offering answers to forensically pertinent questions that genetic analysis alone cannot detect [7] (Figure 2).

Nevertheless, additional investigation is required to confirm the consistency of DNA methylation variations over time, in diverse tissues, using various technologies and methodologies, taking into account also the variability among different populations. Forensic analysis on multiple biological materials found at a crime scene or on evidence such as blood, saliva, hair, skin cells, semen, and tissues can provide useful elements to reconstruct the dynamics of the investigated event and gather comprehensive evidence. Different biological materials may degrade differently over time or under various conditions; hence, analyzing multiple materials ensures a higher chance of obtaining usable evidence, even if some materials are compromised.

In the realm of forensic applications of DNA methylation analysis, one of the primary focuses lies in predicting the biological age of an unidentified donor [13]. Aging is a highly intricate process, encompassing qualitative, quantitative, and inter-individual dimensions [14]. Biologically, aging represents a gradual and inevitable decline in functionality, influenced by the stochastic degradation of its constituent elements, some of which are genetically controlled [15]. Consequently, the cellular and molecular markers of aging are associated with cell senescence, disrupted nutrition sensing, stem cell depletion, creatinine levels, fasting glucose levels, and telomere length [16].

Epigenetic “age estimators” consist of collections of CpGs combined with mathematical algorithms to calculate the age of a DNA source, be it cells, tissues, or organs [17]. The term ‘epigenetic clock’ refers to the progressive age-related DNA methylation alterations occurring at specific genomic locations, remaining consistent across individuals of the same age and enabling age prediction [18] through regularized linear regression models or machine learning techniques by assessing the methylation levels at CpG sites distributed throughout the genome [19,20]. CpGs are sites where guanine linked to a phosphate group follows a cytosine in the 5′-3′ direction. Usually, in mammals, methylated CpGs account for 60–90 percent of all CpGs; on the other hand, unmethylated CpGs are found on CpG islands [21]. The methylation of CpG islands in a gene’s promoter region is generally inversely related to the transcription of that gene owing to the binding of methyl-CpG-binding proteins, which attract proteins to the gene promoter, limiting transcription [22].

There are several techniques available for assessing the DNA methylation levels. Before being analyzed through various forensic methods, DNA should undergo a modification known as bisulfite conversion, enabling the differentiation between methylated and non-methylated cytosines.

There are few studies in the literature dedicated to estimating the biological age of individuals who have left a biological trace. In the field of forensics, as previously noted, a key objective is the precise estimation of age. When the identity of a perpetrator is unknown, predicting age can assist in solving a crime by effectively reducing the range of potential suspects. This research sought to ascertain the ages of various individuals based on human biological samples obtained from Italian Caucasian volunteers. The primary goal of our project was to play a role in establishing standardized protocols for DNA methylation analysis and interpretation patterns specifically tailored for forensic applications in the Italian context. Thus, this study aimed to evaluate the efficacy of a five-gene (ELOVL2, FHL2, KLF14, MIR29B2C, and TRIM59) methylation rate analysis in predicting the age of different individuals using human buccal swabs collected from a sample of 60 Italian volunteers, free from chronic and/or degenerative diseases, with a negative history of smoking, and/or alcohol abuse/dependence, and/or drug addiction.

## 2. Results

All the samples were analyzed following the protocol described in Section 4. The age of the enrolled volunteers, in the present study, was from 23 to 70 years. As previously specified, the methylation levels in the five polymorphisms considered were analyzed. DNA from each sample underwent dual bisulfite conversion, followed by two amplifications for each converted eluate, generating four replicates per sample. Subsequently, the four amplification products underwent identical downstream processes, including the repetition of the single-base extension (SBE) step. After capillary electrophoresis, the methylation levels at specific loci (ELOVL2, FHL2, KLF14, C1orf132, and TRIM59) were calculated and initially input into the tool developed by Onofri et al. [4]. Given that the model proposed for other populations [5] had already been demonstrated to be not applicable to the Italian population [4], we firstly tried to assess whether regression models previously obtained for blood samples from an Italian population by Onofri et al. were also applicable when using buccal swab samples [4]. Two-tail t-tests assuming equal variance applied to the data of each locus revealed no significant heterogeneity between the peak-ratio means of the two conversion reactions; therefore, the average of the replicates was calculated for each sample. The results obtained highlighted an increased predicted age compared to the chronological age of each individual. The comparison among the obtained data showed an MAD (mean absolute deviation) of approximately 10.28 and 13.07 years for the five-gene prediction model (ELOVL2, FHL2, KLF14, C1orf132, TRIM59) and the four-gene prediction model (ELOVL2, FHL2, KLF14, C1orf132), respectively. In light of these results, the development of a new specific tool for buccal swab analysis became necessary. Subsequently, we randomly divided the methylation profiles of the 60 volunteers into two equally sized groups (30 individuals each). One group served as the training set, used for creating a multivariate linear regression predictive model, while the other served as the test set.

**Figure 3:**
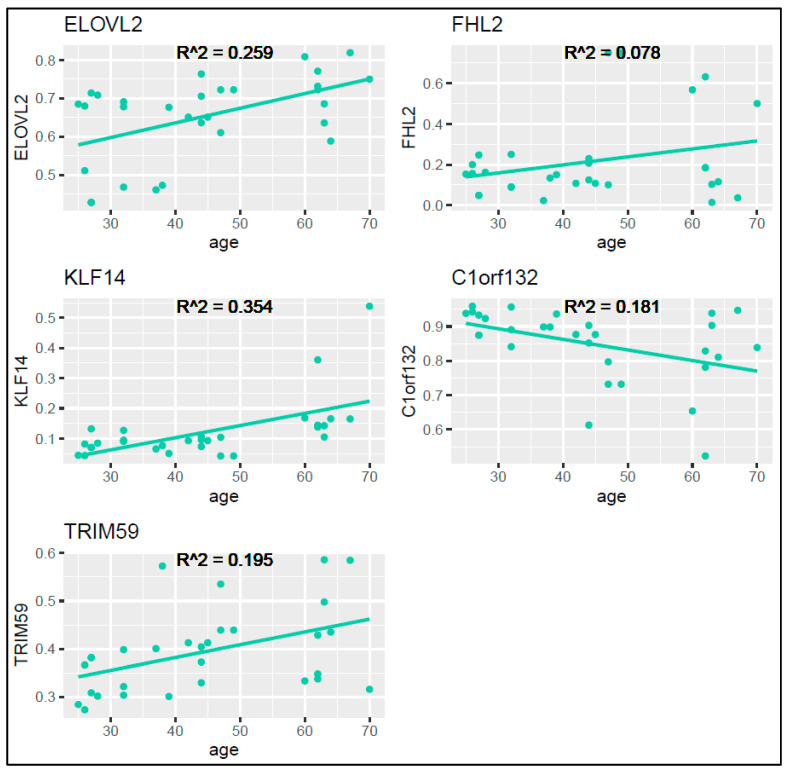
Scatterplots illustrating the relationship between the chronological age and the DNA methylation levels at each of the five examined loci for the training set. Each green point represents a sample, and each line represents the linear regression for each locus.

For the training set, we utilized the ggplot2 package in R to graphically represent the methylation levels against the chronological age for each locus (Figure 3). Pearson correlation coefficients resulted to be moderate or low, in particular, 0.509 for ELOVL2, 0.280 for FHL2, 0.595 for KLF14, 0.425 for C1orf132, and 0.441 for TRIM59, with the R^2^ correlation coefficients as displayed in Figure 3.

A multivariate linear regression model was crafted on the training set. Subsequently, the test set was employed to validate the predictive model obtained from the training set results. The multivariate predictive model facilitated the estimation of the test individuals’ predicted age based on the methylation pattern of each gene, as determined by the following formula:

Predicted age (in years) = 50.04231273 × ELOVL2 DNA methylation level − 11.16979445 × FHL2 DNA methylation level + 73.87032325 × KLF14 DNA methylation level − 49.27304295 × C1orf132/MIR29B2C DNA methylation level + 84.17900486 × TRIM59 DNA methylation level + 13.96381427

**Figure 4:**
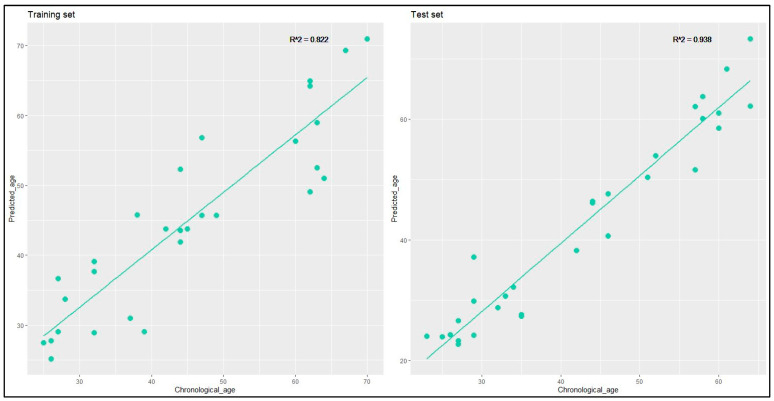
Scatterplots illustrating the correlation between the chronological age and the predicted age of each individual, for the training and the test sets of buccal swab samples. Each green point represents a sample, and each line represents the linear regression between the chronological age and the predicted age.

The relationship between predicted age and chronological age was graphed for the training and the test set (Figure 4), revealing highly robust correlations, with an R^2^ coefficient of 0.822 for the training set and 0.938 for the test set.

The model predictive accuracy was evaluated for the test set by computing the MAD, yielding values of 2.73 for the 23–30 years age group, 2.42 for the 31–40 years age group, 3.16 for the 41–50 years age group, 2.67 for the 51–60 years age group, and 4.52 for the 61–70 years age group. The overall MAD value, encompassing all age groups, was determined to be 3.49.

## 3. Discussion

The analysis of DNA methylation patterns in forensic genetics offers a range of applications, providing valuable information for individual identification, tissue differentiation, age estimation, fluid identification, postmortem interval determination, and environmental exposure assessment.

In our study, we initially assessed the applicability of the predictive models already proposed for blood samples collected from volunteers belonging to the same population (Italians). This was done to verify whether the models suggested by Onofri et al. (inclusive of four and five genes) for blood samples could be universally applied, regardless of the biological material considered [4]. This project aims to contribute to the standardization of DNA methylation analysis protocols for forensic applications in Italy. It is suggested that practical applications may include defining guidelines and standard protocols for DNA methylation analysis in forensic settings. The results obtained indicated a significant overestimation of the predicted age for both the four-gene and the five-gene models, with values approximately 67 years higher than the chronological age for the five-gene model and approximately 77 years higher for the four-gene model. Additionally, we observed an MAD of 10.28 for the five-gene model and of 13.07 for the four-gene model. These findings highlight that the predictive models initially proposed for blood samples may not be universally applicable to saliva samples. This underscores the importance of adapting predictive models to the specific characteristics of the biological material under examination, such as saliva samples. This consideration is relevant for practical applications in forensic contexts, where biological samples can vary. Consequently, we proceeded to divide our dataset into 30 randomly selected samples for the training set to construct the regression model and 30 samples for the test set for model validation.

Therefore, to decipher the complex associations between the methylation levels of distinct genes and the predicted age, our research utilized both individual gene regression analysis and a multiple regression approach (inclusive of the five genes). The multiple regression analysis conducted on our dataset aimed to unravel the relationships between age and the methylation levels of different genes. The multiple R value of approximately 0.91 for the training set indicated a very strong correlation between the dependent and the independent variables. In the same context, the R-squared value obtained was approximately of 0.82, suggesting that about 82% of the variability in the predicted age was explained by the multiple regression model. This implied that the variability in age observed could be largely explained by the combined action of numerous genes.

Nevertheless, when we delved into the analysis of individual genes using linear regression, an interesting observation emerged. The R-squared values for these individual gene regressions were notably lower than those for the overall model, presenting a potential discrepancy. Several factors might have contributed to this phenomenon. First, each gene might respond differently to aging, and capturing this variability in the regression of a single gene can be complex. It is known that biological processes are intrinsically complex, and the influence of various factors on individual genes may not be fully exhausted in isolated analyses.

A more comprehensive view of the intricate relationships within the dataset was provided by the multiple regression model, which also considered the interactions between the chosen genes. Although individual genes’ R-squared values are smaller, the collective effect of several genes may significantly increase the model’s overall explanatory power. Similarly, there is a chance that overfitting will occur when analyzing individual genes, particularly when there are few observations. This could lead to models that overfit a particular dataset but struggle to be generalized to other data. Given that the R-squared values of the individual gene regression analyses in this study could be lower, it is critical to interpret these findings within the framework of the multiple regression model. A worldwide predictive strategy is consequently necessary due to the intricacy of the gene–gene interactions and the impact of aging on them.

Furthermore, the model’s predicted output for each observation exhibited a strong correlation with the corresponding chronological age of the sample. Within the test set, a robust correlation was evident, underscoring the impact of the individuals’ chronological age on the multivariate linear regression model used for age estimation. The prediction accuracy of the model applied, assessed through the mean absolute deviation (MAD), was examined for the test dataset, revealing a prediction error of 3.49 years. This value is lower than that reported in a previous study using a two-gene predictive model (ELOVL2, EDARADD), where a test set of 50 saliva samples showed an MAD of 6.25 years [23], and slightly lower than that reported by Jung et al., of 3.55 years [5]. Notably, when categorizing samples into age groups, the MAD was fairly consistent across different age groups, except for the 61–70 years age group, aligning with an increase in the chronological age of the samples. The observed occurrence might be connected to an increased diversity in methylation levels among older individuals, arising from the cumulative influences of environmental factors, stressors, and lifestyle habits that favor methylation. This study has the potential to influence the development of future investigative and diagnostic tools by providing insights into the complexities of DNA methylation patterns and their applications in forensic age estimation. The findings may contribute to more accurate and adaptable tools for forensic investigations and age-related diagnostics. The study’s approach of using DNA methylation patterns could enhance the precision of age predictions, aiding in criminal investigations where accurate age determination is crucial.

The main limitation of this study is the small sample size, comprising only 60 participants, which might constrain the generalizability of the findings and which, if expanded, could yield more robust results in the context of regression lines, especially linear ones. The awareness of limitations is essential for interpreting results and guiding the future development of predictive models.

The study has also other limitations. These include the age range, which, although spanning from 23 to 70 years, could be extended to other age groups, especially within the elderly population. The reported Pearson correlation coefficients between methylation levels and chronological age were categorized as low or very low for single genes, suggesting challenges in establishing robust associations. Therefore, while the study makes significant strides in exploring the applicability of predictive models to buccal swab samples, its interpretations should be cautious, considering the acknowledged limitations, which include sample size, age range representation, and challenges in model application and related to the correlation coefficients.

Future perspectives of research in this field may include the possibility of expanding the sample size, potentially including individuals affected by major chronic cardiovascular and metabolic diseases, as well as those with issues of alcohol/drug abuse or addiction. This would allow assessing the utility of incorporating correction coefficients into predictive models or, alternatively, proposing multiparametric age prediction models that take into account not only the methylation profiles but also, for example, other epigenetic modifications.

## 4. Materials and Methods

### 4.1. Sample Collection

**Figure 5:**
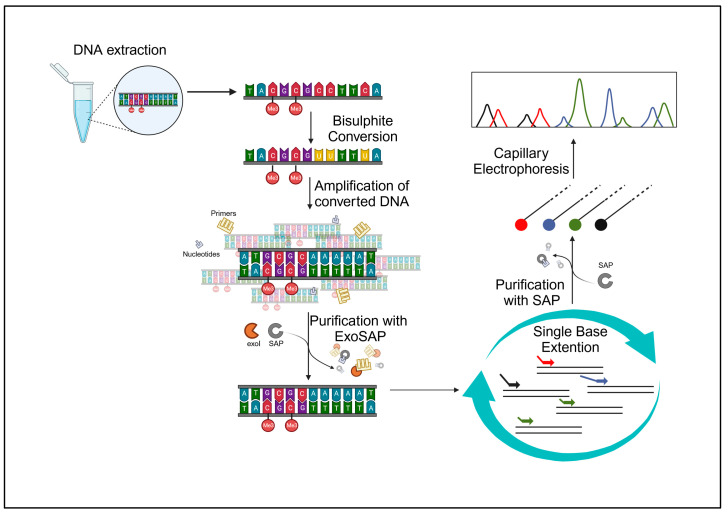
Graphical representation of the experimental workflow (Created with BioRender.com accessed on 20 December 2023).

The study was approved by the Ethics Committee of Perugia University, Umbria, Italy (protocol code 43615, approved on the 23 February 2021). Our samples consisted of 60 buccal swabs, collected from 60 volunteers (30 females and 30 males), aged between 23 and 70 years, free from chronic and/or degenerative diseases, with a negative history of smoking, and/or alcohol abuse/dependence, and/or drug addiction. The volunteers, who had been fully informed about the project beforehand, gathered the samples and willingly contributed their biological specimens anonymously. All specimens underwent anonymization, and solely pertinent data for the study, including chronological age and health/disease status without additional details, were gathered. All the collected samples were analyzed following the experimental protocol described below and shown in Figure 5, followed by a statistical data analysis.

### 4.2. DNA Extraction and Quantification

This method required a series of centrifugation steps combined with the use of enzymatic and chemical reagents provided by the QIAmp^®^ DNA mini kit (Qiagen, Hilden, Germany). The head of each buccal swab was placed in a 2 mL tube with 600 µL of ATL lysis buffer and 20 µL of proteinase K. The process was executed in accordance with the manufacturer’s guidelines.

The extracted DNA was quantified (once per sample) using the Thermo Scientific Nanodrop One Microvolume UV–Vis spectrophotometer, using 1 µL of buffer AE (Qiagen elution buffer) as a “blank”. Then, 1 µL of each sample was loaded in succession, to quantify their respective amount of DNA.

### 4.3. Bisulfite Conversion

For this step, the EZ DNA Methylation-Direct kit (Zymo Research, Irvine, CA, USA) was used, and the protocol provided by the company was followed. The EZ DNA Methylation-Direct™ kit requires a DNA extract input of 20 μL, with a concentration of 250–500 ng/20 μL. The samples’ extracts and controls were diluted or concentrated to obtain a concentration as close as possible to 500 ng/20 μL. Alongside the samples, two control DNAs, one methylated and one non-methylated (HCT116 DKO methylated and non-methylated DNA by Zymo Research) were bisulfite-converted, so that they could be analyzed following the same protocol, in order to check for a correct bisulfite conversion.

### 4.4. PCR Amplification and Cleanup

The PCR primers for the five CpG sites investigated in the genes ELOVL2, FHL2, KLF14, C1orf132, and TRIM59 were as in Jung et al. [5], while the primer mix concentrations and amplification conditions were the ones used by Onofri et al. [4].

The master mix for the amplification was prepared by adding 6.25 µL of QIAGEN Multiplex PCR Master Mix 2x and 1.25 µL of PRIMER MIX amplification 10X to each sample. Then, the master mix was aliquoted in different 0.2 mL tubes, and 4 µL of previously converted DNA samples, since the concentration of the samples had to be 10 ng/4 μL, and 1 µL of water were added in each tube, respectively.

The tubes were loaded in the thermocycler and underwent the following thermal cycle: a first step of denaturation at 95 °C for 10 min; a second step of denaturation, annealing, and amplification at 95 °C for 30 s, 54 °C for 30 s, and 72 °C for 30 s, respectively, repeated for 45 times; and a final extension step at 72 °C for 5 min. The ExoSAP™ Express PCR Product Cleanup reagent (Thermo Fisher Scientific, Waltham, MA, USA) was employed for the enzymatic purification of the amplified product, following the kit manufacturer’s specified conditions for this step. PCR cleanup was carried out for each sample by mixing 2 µL of ExoSAP™ to 5 µL of PCR product.

### 4.5. Multiplex Single-Base Extension and Purification

The multiplex single-base extension (SBE) reaction was performed using the SNaPshotTM Multiplex kit (Thermo Fisher Scientific, Waltham, MA, USA). The SBE primers were those in Jung et al. [5], while the SBE primer mix concentrations were as in Onofri et al. [4].

The SBE reaction per sample was prepared as follows:

- 5 μL of SNaPshot™ Multiplex Ready Reaction Mix;
- 1 μL of SBE PRIMER MIX 10x;
- 1 μL of H_2_O;
- 3 μL of ExoSAP™-purified DNA.
- The conditions for SBE were set, for the 25 cycles, as follows:
- 96 °C for 10 s;
- 50 °C for 5 s;
- 60 °C for 30 s;
- 4 °C until post-SBE purification.

For the post-SBE purification, the kit used was USB^®^ shrimp alkaline phosphatase (SAP) (ThermoFisher Scientific, Waltham, MA, USA). A master mix was prepared with 1 µL of SAP and 2 µL of 10X SAP reaction buffer for each sample, and 5 µL of the SBE product, obtained in the previous step, and 12 µL of water were added in each tube. After this, the samples were incubated at 37 °C for 30 min and then at 65 °C for 15 min.

### 4.6. Capillary Electrophoresis

The analysis of the samples was conducted using a SeqStudio Genetic Analyzer (Thermofisher Sicentific, Foster City, CA, USA). A master mix was prepared by mixing 15 µL of formamide and 0.2 µL of GeneScan™-120 LIZ™ size standard for each sample. The master mix was aliquoted into the appropriate wells of MicroAmp ^®^ Optical 96-well reaction plates. Subsequently, 1.5 µL of each post-SAP SBE product was loaded into the wells, and the plate was placed at 95 °C for the denaturation step for 4 min and then in ice at 4 °C for other 4 min. In the following step, the plate was loaded into the sequencer. The Gene Scan E5 Run module parameters were the same as the ones indicated in the kit protocol, with the only modification of the collection time, which was changed from 24 min to 18 min. For the sample detection, after the electrophoretic run, we utilized the program GeneMapper ID-X v. 1.6 for the analysis of electrophoretic data.

### 4.7. Statistical Analysis

In our case, we chose to calculate the methylation level of each gene using the formula found in the literature in the study by Jung et al. [5].

For the first two polymorphisms (ELOVL2, FHL2), the general formula became:

(1) $$\frac{I_{C}}{I_{C}+I_{T}},$$

whereas, for the other three (KLF14, C1orf132, TRIM59), it became:

(2) $$\frac{I_{G}}{I_{G}+I_{A}}$$

It is possible to consider either peak height or area as intensity (*I*) in this equation. In our case, the calculation was performed considering peak height as *I*. The analysis and graphical representation of the data were conducted using Microsoft® Excel® 2019 (version 16.0.10405.20015), alongside the statistical tools RStudio (version 2023.06.1 Build 524) and Jamovi software (version 2.3) [24,25].

## 5. Conclusions

Nowadays, the analysis of DNA methylation for human age prediction is a method with useful repercussions in forensic investigations, despite its wide application in real cases being still far from being reached.

Our study explored the applicability of predictive models developed for blood samples to buccal swab samples from Italian volunteers. The results indicated a significant overestimation of the predicted age for both four-gene and five-gene models. We developed and validated a regression model, revealing strong correlations in the training set. However, individual genes’ regressions showed lower R-squared values, emphasizing the complexity of biological processes. The model exhibited promising accuracy in predicting age, though challenges were noted for the 61–70 years age group.

While the study provides valuable insights into the applicability of predictive models to buccal swab samples, a careful consideration of its limitations, including the small sample size and the still extendable age range representation, is essential. Overall, the study highlights the importance of a comprehensive approach to understand the interplay of multiple genes in age prediction. It contributes to the understanding of the complex interplay between methylation levels and age, emphasizing the need for a comprehensive predictive approach in the context of forensic genetics. Standardization efforts in DNA methylation analysis protocols can lead to the establishment of guidelines and protocols that will enhance the reliability and consistency of forensic analyses, potentially influencing future investigative practices.

## Data Availability

The data presented in this study are available on request from the corresponding author.

## References

[1] Kayser M., Branicki W., Parson W., Phillips C. (2023). Recent Advances in Forensic DNA Phenotyping of Appearance, Ancestry and Age. Forensic Sci. Int. Genet..

[2] Dabas P., Jain S., Khajuria H., Nayak B.P. (2022). Forensic DNA Phenotyping: Inferring Phenotypic Traits from Crime Scene DNA. J. Forensic Leg. Med..

[3] Tozzo P., Politi C., Delicati A., Gabbin A., Caenazzo L. (2021). External Visible Characteristics Prediction through SNPs Analysis in the Forensic Setting: A Review. Front. Biosci..

[4] Onofri M., Delicati A., Marcante B., Carlini L., Alessandrini F., Tozzo P., Carnevali E. (2023). Forensic Age Estimation through a DNA Methylation-Based Age Prediction Model in the Italian Population: A Pilot Study. Int. J. Mol. Sci..

[5] Jung S.-E., Lim S.M., Hong S.R., Lee E.H., Shin K.-J., Lee H.Y. (2019). DNA Methylation of the ELOVL2, FHL2, KLF14, C1orf132/MIR29B2C, and TRIM59 Genes for Age Prediction from Blood, Saliva, and Buccal Swab Samples. Forensic Sci. Int. Genet..

[6] Soto-Palma C., Niedernhofer L.J., Faulk C.D., Dong X. (2022). Epigenetics, DNA Damage, and Aging. J. Clin. Investig..

[7] Sabeeha S., Hasnain S.E. (2019). Forensic Epigenetic Analysis: The Path Ahead. Med. Princ. Pract..

[8] Haddrill P.R. (2021). Developments in Forensic DNA Analysis. Emerg. Top. Life Sci..

[9] Kader F., Ghai M. (2015). DNA Methylation and Application in Forensic Sciences. Forensic Sci. Int..

[10] Konrad H., Jürgens L., Hartung B., Poetsch M. (2023). More than Just Blood, Saliva, or Sperm—Setup of a Workflow for Body Fluid Identification by DNA Methylation Analysis. Int. J. Legal Med..

[11] Kader F., Ghai M., Olaniran A.O. (2020). Characterization of DNA Methylation-Based Markers for Human Body Fluid Identification in Forensics: A Critical Review. Int. J. Legal Med..

[12] Vidaki A., Kayser M. (2018). Recent Progress, Methods and Perspectives in Forensic Epigenetics. Forensic Sci. Int. Genet..

[13] Noroozi R., Ghafouri-Fard S., Pisarek A., Rudnicka J., Spólnicka M., Branicki W., Taheri M., Pośpiech E. (2021). DNA Methylation-Based Age Clocks: From Age Prediction to Age Reversion. Ageing Res. Rev..

[14] Field A.E., Robertson N.A., Wang T., Havas A., Ideker T., Adams P.D. (2018). DNA Methylation Clocks in Aging: Categories, Causes, and Consequences. Mol. Cell.

[15] Zhang J., Wang S., Liu B. (2023). New Insights into the Genetics and Epigenetics of Aging Plasticity. Genes.

[16] Bell C.G., Lowe R., Adams P.D., Baccarelli A.A., Beck S., Bell J.T., Christensen B.C., Gladyshev V.N., Heijmans B.T., Horvath S. (2019). DNA Methylation Aging Clocks: Challenges and Recommendations. Genome Biol..

[17] Horvath S., Raj K. (2018). DNA Methylation-Based Biomarkers and the Epigenetic Clock Theory of Ageing. Nat. Rev. Genet..

[18] Tan Q., Heijmans B.T., Hjelmborg J.V.B., Soerensen M., Christensen K., Christiansen L. (2016). Epigenetic Drift in the Aging Genome: A Ten-Year Follow-up in an Elderly Twin Cohort. Int. J. Epidemiol..

[19] Aliferi A., Ballard D., Gallidabino M.D., Thurtle H., Barron L., Syndercombe Court D. (2018). DNA Methylation-Based Age Prediction Using Massively Parallel Sequencing Data and Multiple Machine Learning Models. Forensic Sci. Int. Genet..

[20] Fan H., Xie Q., Zhang Z., Wang J., Chen X., Qiu P. (2022). Chronological Age Prediction: Developmental Evaluation of DNA Methylation-Based Machine Learning Models. Front. Bioeng. Biotechnol..

[21] Vidaki A., Daniel B., Court D.S. (2013). Forensic DNA Methylation Profiling—Potential Opportunities and Challenges. Forensic Sci. Int. Genet..

[22] Tammen S.A., Friso S., Choi S.-W. (2013). Epigenetics: The Link between Nature and Nurture. Mol. Asp. Med..

[23] Hamano Y., Manabe S., Morimoto C., Fujimoto S., Tamaki K. (2017). Forensic Age Prediction for Saliva Samples Using Methylation-Sensitive High Resolution Melting: Exploratory Application for Cigarette Butts. Sci. Rep..

[24] Posit Team (2023). R Studio: Integrated Development Environment for R.

[25] The Jamovi Project. *Jamovi [Computer Software]*, Version 2.3; Sydney, Australia, 2022. https://www.jamovi.org.

